# The early impact of the Covid-19 pandemic on the global and Turkish economy

**DOI:** 10.3906/sag-2004-6

**Published:** 2020-04-21

**Authors:** Ömer AÇIKGÖZ, Aslı GÜNAY

**Affiliations:** 1 Department of Economics, Faculty of Political Sciences, Social Sciences University of Ankara, Ankara Turkey

**Keywords:** Covid-19, economy, pandemic

## Abstract

**Background/aim:**

Individuals infected by the Covid-19 potentially are at risk of health and economic well-being. Today, the Covid-19 is a global issue, and the world economy can be interpreted as almost at the standstill. In this context, this study aims to discuss the potential first reactions of short and long term global economic impacts of the pandemic through sectors by assessing its costs according to the data announced for both the world and Turkey. In addition, this study tries to put forth possible economic and political scenarios for the post-pandemic world.

**Materials and methods:**

This is a review article that summarizes the current reports and discussions about the economic consequences of this historical event, and tries to make some inferences considering them.

**Results:**

This pandemic has severe adverse effects on the employees, customers, supply chains and financial markets, in brief, most probably it will cause a global economic recession. Nevertheless, due to the uncertainty of the end of this pandemic, both the length and scale of this contraction are not predictable.

**Conclusion:**

It takes a while for the world economy to recover from the contraction. It seems that this pandemic will lead to a permanent shift in the world and its politics, especially in health, security, trade, employment, agriculture, manufacturing goods production and science policies. Since this new world might provide great opportunities for some countries that did not dominate world production before, governments should develop new strategies to adjust the new world order without much delay.

## 1. Introduction

Scientists in the field express that Coronaviruses (CoVs) are a large family of viruses that cause illness ranging from the common cold to more severe diseases such as Middle East respiratory syndrome (MERS-CoV) and severe acute respiratory syndrome (SARS-CoV). The Coronavirus disease (Covid-19) is a new strain that was discovered in China in 2019 and has not been previously identified in humans and earth. As of 7 April 2020, the Covid-19 had infected more than 1.2 million people, killed more than 72 thousand and spread all over the world [1]. The latest situation has updated by the World Health Organization (WHO) day by day. 

On 11 March 2020, the WHO officially declared the Covid-19 outbreak as a pandemic due to the global spread and severity of the disease. Before the Covid-19, there are four pandemics in the last two century: “Spanish Flu” [caused by an Influenza A(H1N1) virus] in 1918, the “Asian Flu” [caused by an Influenza A(H2N2) virus] in 1957, and the “Hong Kong Flu” [caused by an Influenza A(H3N2) virus] in 1968 plus swine flu in 2009. Pandemic comes etymologically from a Greek word “pandemos”, it means a global epidemic, it is distinguished from an epidemic, which may connote limitation to a smaller area and a concept pertaining to all people and the public in the world. Therefore, a pandemic is defined as the highest level of global health emergency and affecting multiple regions of the world. In general, declaring a pandemic is assumed a historical event since it is not just a health issue, it has also economic, political and social dimensions at the global level. 

If social, political and economic life in the future will show different trends after the Covid-19 as predicted, the future of cities with populations of 30 million will be questioned further. The values of collective living of traditional societies that resist modernism will become more controversial. While the speed of individualization which begins with modernism will increase, nation-states might feel themselves under more threat. Groups that have the same sentimental values as religious communities will lose their momentum and they will begin to transform into an individual nature as well. In addition, multi-storey buildings and crowded cities will lose their speed of development, the existing clean water networks and the apartment life where the community lives together will become questionable due to the threat of life safety. As crowded cities will tend to lose their significance achieved after the industrial revolution, cleanliness, health, and hygiene will have a priority in social life. Moreover, both communities and countries might disrupt some democratic values and fundamental human rights; hence, authoritarianism might become widespread by increased security measures. The future of healthcare sector, the personal and professional rights of its professionals, ethic values like physicians right to refuse medical treatment for life-threatening illness or life-sustaining, and also the establishment of hospitals for untreated illness will be among the main issues to be discussed soon but most probably these future planning will cost the world economy. 

Old habits related to consuming unpackaged foods and sales market conditions will not be able to pursue as it is, and new protection mechanisms such as preservation and packaging for the nutritious food will bring new costs to the consumers. With the very high concern for cleanliness and health, the future of paper money and coins circulated for the transactions will also be discussed further, and governments will create new customs walls. Hence, countries’ trade amount and speed will be under threat, and countries ensuring the hygiene standard comparing to others will gain a competitive advantage. On the other hand, global viruses will be seen as a threat like a nuclear attack or global terrorism since their impacts on the human and the world are similarly very destructive as seen in the Covid-19. For this reason, countries that could develop a biological defence system or infrastructure for the virus attack will have more power in the world. However, if they will use this power to achieve their national and imperial goals, this will most likely create even more inequality among the countries.

Today, the spreading of the Covid-19 virus is having gradual effects like major economic depression. Economically, if countries hit by sudden and unexpected disease, countries’ revenue will drop due to the fall in overall economic activities. The contraction of the economy would begin with the diminishing of the travel and tourism sectors. Then, their widespread effects damage aggregate production and consumption with the layoffs and bankruptcies in all sectors. The unemployment rate rises, and to survive the economy, governments and the central banks must step in by increasing government spending and decreasing interest rates, respectively to increase consumer demand and investments. Nevertheless, estimating the economic costs from a global disease is now ambiguous since the pandemic has spiral effects both on the national and global economy that means any economic shock to one country is quickly spread to other countries through the increased trade and financial linkages associated with globalization [2]. According to the World Bank (WB) report published in 2013, “… a severe pandemic would resemble a global war in its sudden, profound, and widespread impact,” and a severe pandemic could cause economic losses equal to nearly 5% of global Gross Domestic Product (GDP), or more than $3 trillion [3]. The United Nations Conference on Trade and Development (UNCTAD) announced that the Covid-19 will likely cost the global economy at least $2 trillion in 2020 [4]. 

In accordance with the predictions presented above, especially because of their economic contents, it is necessary to investigate the impact of the Covid-19 pandemic on the global and Turkish economy. Some studies in the literature are examining the global or regional economic costs of diseases such as SARS [2,5–8]. In addition, the World Bank and the Rockefeller Foundation published a report about pandemic risk even long before the pandemic [3,9]. Recently, the media and some foundations have reported several reports on the economic consequences of the Covid-19 pandemic but so far, there has been no study analysing the potential consequences of a pandemic on the world and the Turkish economy in the literature yet. In this context, the primary purpose of this study is to fill this gap by presenting the likely macroeconomic effects of the latest pandemic according to the data announced for both the world and Turkey. Besides, this study aims to forecast the possible short and long term effects of the pandemic on the global economy and politics by taking into account the current discussions.

## 2. Global economic costs of pandemic

China, where the Covid-19 virus originated in, is the most populous country with nearly 1.4 billion residents^1^1World Bank (2018). World Bank Open Data: Population, total [online]. Website https://data.worldbank.org [accessed 25 March 2020].and the world’s second biggest economy, with a GDP of $13.6 trillion^2^2World Bank (2018). World Bank Open Data: Gross Domestic Production (current US$) [online]. Websitehttps://data.worldbank.org [accessed 25 March 2020].
China has been fighting the Covid-19 pandemic since December 2019 but this battle devastated China’s economy in the first quarter of 2020. China is the crucial country as a source of both demand and supply, and a focus of concern for financial markets for the rest of the world; hereby, adverse developments in China have spillover effects on the other countries [10]. The Organisation for Economic Co-operation and Development (OECD) announced that growth prospect for China has been revised down sharply to below 5% this year after 6.1% in 2019. Accordingly, global economic growth is projected to fall to 2.4% in 2020, compared to 2.9% in 2019, but it could fall as low as 1.5% due to the drop in overall global economic activities [10]. 

Tourism is a huge global business that accounts for 10.4% of global GDP and 10% of global employment according to the World Travel & Tourism Council (WTTC) [11]. Only Chinese tourists account for around one-tenth of all cross-border visitors and a huge drop in outbound tourism from China leads to an adverse demand shock in many countries [10]. With the spreading of the virus all around the world, the World Tourism Organization (UNWTO) announced that the tourism sector is currently one of the hardest-hit by the Covid-19, with impacts on both travel supply and demand. An expected fall in international tourist arrivals will be between 20% and 30% could translate into a decline in international tourism receipts of between $300 and 450 billion [12]. The International Air Transport Association (IATA) estimated cost airlines as much as $252 billion with a 44% drop in lost revenue due to the collapse of the airline industry [13].

On the other hand, China ranked first in goods and services exports with an export value of about $2.7 trillion and second in imports with $2.6 trillion in 2018^3^3World Bank (2018). World Bank Open Data: Exports and Imports of Goods and Services (current US$) [online]. Website https://data. worldbank.org [accessed 25 March 2020].. Accordingly, China’s production relations with other countries cover a broad space that cannot be filled quickly. China has a key role in global supply chains as a producer of intermediate goods, particularly in computers, electronics, pharmaceuticals, and transport equipment, and as the primary source of demand for many commodities. General Administration of Customs stated that China’s overall exports and imports contracted by 17.2% and 4%, respectively in the first two mounts of 2020 [14]. Over that time, China’s industrial production fell by 13.5% and industrial profits decreased by 38.3% [15,16]. Around the world, businesses are dealing with lost revenue and disrupted supply chains due to factory closures in China. However, the extended factory closures in China will not only affect the supply of those products but also hurt other markets’ ability to produce goods. 

Furthermore, the uncertainty in China is harmful to global oil prices since China is the world’s biggest oil importer with 20.2% of total crude oil imports^4^4World’s Top Exports (2018). Crude Oil Exports by Country [online]. Website http://www.worldstopexports.com [accessed 26 March 2020].. Nevertheless, global oil demand has been hit hard by the Covid-19 because of the widespread contraction of China’s economy and the drop in demand fuels and gasoline with the travel bans. Meanwhile, the price war between major oil producers Saudi Arabia and Russia continues, and they have continuously increased production to overwhelm global oil markets with supply. The price of something is determined by the interplay of supply and demand but two of the world’s biggest oil producers cause a huge unbalance between demand and supply in the oil market. As a result of this, oil prices lost as much as a third of their value since the 1991 Gulf War^5^5Reuters (2020). Oil Plunges 25%, Hit by Erupting Saudi-Russia Oil Price War [online]. Website https://www.reuters.com [accessed 23 March 2020].. In theory, falling oil prices should be good for growth since business costs fall when fuel becomes cheaper and consumers’ purchasing power increase for oil-based energy products. However, there is unlikely to be much of fuel demand in the short term due to the wide-ranging travel bans and curfews. 

The International Labour Organization (ILO) announced that between 5.3 million and 24.7 million jobs will be lost because of the economic crisis caused by the Covid-19, and this deterioration in employment also means a large loss of income between $860 billion to $3.4 trillion by the end of 2020 for workers [17]. For example, in March 2020, the unemployment rate increased by 0.9% point to 4.4% in the United States (US) [18] and more than 6.6 million Americans have filed unemployment claims since the pandemic began in the United States (US) [19]. 

Global stock markets have fallen sharply as investors continue to worry about the broader uncertain economic effects of the pandemic. The FTSE, Dow Jones Industrial Average and the Nikkei have all seen huge falls (more than 25%) since the pandemic began^6^6BBC News (2020). Coronavirus: A Visual Guide to The Economic Impact [online]. Website u2990[accessed 24 March 2020].. Net portfolio flows and the value of currencies of emerging economies against the dollar have dropped significantly since the beginning of 2020 [20].To calm markets and encourage spending, central banks in many countries have cut interest rates to make borrowing cheaper and injected liquidity in the financial market to guarantee liquidity in sovereign and private credit markets. For example, the US Federal Reserve and the Bank of England cut their key interest rate to near zero 77The Guardian (2020). Federal Reserve Cuts Interest Rates to Near Zero in Attempt to Prop up US Economy [online]. Website https://www.theguardian.com [accessed 23 March 2020]..In addition, the European Central Bank (ECB) took action, spending a €750 billion buying government debt and private securities before the end of 2020 [21]. 

Recently, governments have started to provide a sizable economic support package for their citizens and businesses, including mainly wage subsidies, cash transfers to low-income households, as well as tax cuts and rent reductions for businesses. Also, both the International Monetary Fund (IMF) and the WB announced $50 billion and $14 billion packages, respectively for financing to help countries suffering from the pandemic [22,23]. In addition, the US Senate passes a historic $2.2 trillion rescue package and the United Kingdom (UK) pays up to 80% of employee wages for those unable to work due to the pandemic to prevent bankruptcies, massive layoffs and negative impact on aggregate demand^8^8World Economic Forum (2020). The Economic Effects of COVID-19 Around The World [online]. Website https://www.weforum.org [accessed 3 April 2020]..

Economists predict that the pandemic is putting downward pressure on inflation due to the volatility in supply and demand shocks working in opposite and asynchronous ways [24]. While China’s Consumer Price Index (CPI) rose by 5.2% due to mainly food inflation, the Producer Price Index (PPI) dropped 0.3 % in February due to mainly decline in the price of raw materials [25,26]. Also, consumers intended to increase their spending on groceries and non-food products for children in the US in March 2020 and they expected to decrease their spending for almost every other product category^9^9Statista (2020). Statistics and Facts on Retail & Trade: Impact of Coronavirus (COVID-19) on Consumer Spending in The United States [online]. Website https://www.statista.com [accessed 2 April 2020].. Therefore, inflation in other countries will likely follow a similar trend like China and the impact on consumer prices will continue until the production starts again. UNCTAD estimated the overall price decline would be 37% in 2020 [20]. 

According to the United Nations Educational, Scientific and Cultural Organization (UNESCO) monitoring, over 160 countries have implemented nationwide closures, involving over 91% of the world’s student population^10^10United Nations Educational, Scientific and Cultural Organization (2020). Covid-19 Impact on Education [online]. Website https:// en.unesco.org [accessed 3 April 2020].. It carries high economic costs since working parents are more likely to miss work that leads to a wage loss and adverse impact on production in the short term. In the long term, this situation will bring a deterioration in human capital that has a great impact on the economic development of countries. 

## 3. Costs of pandemic on the Turkish economy

Turkey had managed to keep the Covid-19 virus away from the country until 11 March 2020 when the first confirmed case was declared. Before that, the OECD increased its expectation for Turkish economy growth to 2.7% from 0.9% and predicted that this pandemic would have a positive impact on the Turkish economy if the government would take well-focused policy measures [10]. Accordingly, Turkey expected the tourism boom and global demand shift in some sectors such as textiles, furniture, iron and steel, and food. However, these predictions will not come true at least in the short term since pandemic has spread throughout the country and all around the world, and now Turkey estimates the potential economic costs of this crisis.

Turkey is the 19th largest economy in the world, with a GDP of $771 billion2. Turkey’s overall export and import values were nearly $171 billion and $202 billion, respectively resulting in a negative trade balance of $31 billion in 2019. The top export destinations of Turkey are Germany (9%), UK (6.3%), Italy (5.4%), Iraq (5.2%) and the US (4.7%) and the top import origins are Russia (11.1%), China (9.1%), Germany (8.9%), the US (5.5%) and Italy (4.2%) [27], these data show that the supply chain of Turkey does not depend on China largely but other countries’ economies have also hit by the pandemic; hence, this would hurt the Turkish manufacturing sector in the end. In March 2020; exports decreased by 17.81% and imports increased by 3.13% compared with March 2019 due to the pandemic [28]. Turkish current account deficit recorded $1.804 million indicating an increase of $1.528 million compared to January of the previous year, bringing the 12-month rolling surplus to $6.494 million [29].However, the decline will be expected in 2020 due to a lower goods trade surplus, which results from the global economic disruptions caused by the pandemic.

Turkey has struggled with the high unemployment rate (13.7%) since 2018 [30]. It seems that the unemployment rate will increase especially among the blue-collar workers and service sector employees due to the bankruptcies, and closures of factories and workplaces, who will probably suffer the first economic losses of this pandemic. This will lead to a drop in the GDP growth of Turkey and income losses for workers. Besides, the CPI of Turkey increased by 11.86% annually and 0.57% monthly in March 2020. Health with 2.78%, food and nonalcoholic beverages with 1.95% and miscellaneous goods and services with 1.24% were the main groups where high monthly increases realized [31]. Therefore, the rise will be expected at Turkish consumer prices through food inflation in the short term like all around the world. In addition, Turkey had around 50 million visitors and $34.5 billion of tourism income in 2019 [32], but the tourism sector will be hardly damaged if the pandemic will not end by this summer.

Additionally, uncertainty will bring more risks for investors. Turkey’s Economic Confidence Index fell from 97.5 to 91.8 [33] and the Real Sector Confidence Index decreased from 106.9 to 99.7 [34] in March 2020 compared to the previous month due to the pandemic. The Central Bank of the Republic of Turkey (CBRT) reduced the inflation rate from 10.75% to 9.75% to improve the financial conditions [35]. Today, Turkey’s 5 Years Credit Default Swap (CDS) premium value is high with 652.3 points on 7 April 2020 compared to 230 points at the beginning of 2020^11^11World Government Bonds (2020). Turkey 5 Years CDS-Historical Data [online]. Website http://www.worldgovernmentbonds.com [accessed 7 April 2020]. , and it can be interpreted that Turkish economy is under the pressure of financial risk. Therefore, this higher CDS premium might cause pressure on the Turkish foreign borrowing interest rate to rise. 

The Turkish government announced a $15.4 billion economic stimulus package on 18 March 2020^12^12Bloomberg (2020). Turkey Unveils $15.4 Billion Plan to Counter Virus Outbreak [online]. https://www.bloomberg.com [accessed 1 April 2020]., introducing a mix of tax cuts, payment deferrals and increased pension payouts to help citizens and businesses. However, it seems that the government must pursue a more long term economic measures to save jobs, reassure businesses and lessen the burden on those who have left work. Turkey is also hosting the largest refugee population in the world, mostly Syrians displaced by the neighboring civil war^13^13 UN Refugee Agency (2020). Refugee Population Data [online]. Website https://www.unhcr.org/data.html [accessed 2 April 2020]. and this will bring a financial burden to the government to protect these people. Nevertheless, if the oil prices would low in the long term, Turkey’s petroleum bill will decrease, and this might be life-saving for the Turkish economy as an energy importer country.

## 4. Discussion

The OECD warned that the shock from the virus is already bigger than the 2008 global financial crisis.The OECD Secretary General Angel Gurría said: “many countries would fall into recession and countries would be dealing with the economic fallout of the Covid-19 pandemic for years to come”^14^14BBC News (2020). Global Economy Will Suffer for Years to Come, Says OECD [online]. Website https://www.bbc.com [accessed 24 March 2020].. In addition, the IMF Managing Director Kristalina Georgieva stated that “the outlook for global growth: for 2020 it is negative–a recession at least as bad as during the global financial crisis or worse. However, we expect a recovery in 2021. To get there, it is paramount to prioritize containment and strengthen health systems–everywhere” [36]. According to these statements, we can say that quicker recovery will depend on the uncertain time of the end of the pandemic in the world.

According to the McKinsey & Company report published in March 2020, the world economy will face two possible future scenarios: delayed recovery or prolonged contraction. In the first scenario, unemployment will rise; bankruptcies will put pressure on the banking system; more liquidity and low interest rate will have a limited effect on demand; self-reinforcing recession will extend, and recovery will begin in the fourth quarter of 2020. In the second scenario, increasing layoffs and bankruptcies will feed to a self-reinforcing recession; a comprehensive banking crisis will damage the world financial system, and all fiscal and monetary responses will be insufficient to stop the recession until the second quarter of 2021 [37].

The OECD, IMF and World Bank have already warned the governments to take effective and well-resourced public health measures and implement well-targeted policies to support health care systems to overcome the Covid-19 crisis [10,22,23]. Moreover, they have forced governments to take supportive macroeconomic and fiscal measures to restore confidence and demand in the economy, and protect the jobs and the incomes of workers and businesses. Member of G20 governments announced in March 2020 that they will focus mainly on safeguard people’s jobs and incomes, restore confidence, preserve financial stability, and minimize disruptions to trade and global supply chains with global cooperation [38]. Nevertheless, all support mechanisms will bring an extra burden on public economics, and most probably, the public debt will increase more with the decreasing tax revenues. 

The Covid-19 pandemic led to not only the public health crisis but also combined with a global recession, it has the potential to change politics and power around the world. This is the big historical event like the fall of the Berlin Wall, Brexit and September 11 Attacks for the world. According to the twelve leading global thinkers’ predictions^15^15Foreign Policy (2020). How The World Will Look After The Coronavirus Pandemic. [online].Website https://foreignpolicy.com [accessed 1 April 2020]., the pandemic will change the world forever. They pointed out that this pandemic is a world-shattering event whose far-ranging consequences we can only begin to imagine today. For example, it will strengthen the state, reinforce nationalism, change global economic directions between East and West, and a new stage in globalization will be started by the changing supply chain production strategies. Additionally, American power might be criticized; some countries might fail, and countries might prefer stability to profitability. 

**Table 1:** Threats and opportunities for the post-pandemic world.

Threats	Opportunities
• Prolonged contraction • Bankruptcies and higher unemployment rate • Insufficient supply chain • Volatility in oil prices • Huge drop in consumer spending and business investment • Banking crisis • Huge public deficits • More travel restrictions • More custom restrictions • Food inflation • More failed countries • Trade policies	• More workforce protection • New working conditions • New supply chain mechanisms • More online shopping • More digitalization • Emerging of new trade mechanisms • Changing in expenditure habits • More hygiene and safety rather than more profits • Helping each other • Multilateralism • Social awareness of clean environment • New and smart technologies • Fruitful and cost-efficient meetings

Before the Covid-19, the report published by the Rockefeller Foundation in 2010 [9] stated that governments would impose strict rules and restrictions on the public during and even after the pandemic to protect themselves. At first, the notion of a more controlled world would gain wide acceptance and approval. Then, the economic models conducted by authoritarian leaders would increase but authoritarianism greatly would restrict entrepreneurial activity and innovation. Hence, the public would support more restrictions and control mechanisms but this would lead to deteriorations of the fundamental citizens’ rights. Moreover, the health and freedom paradigm might be one of the top topics in addition to the security and freedom paradigm in public policy, and authoritarianism and co-operation will be discussed as two opposite approaches to overcome the health crisis in the post-pandemic world. 

Currently, the Covid-19 brings some new issues in labour and production markets. For example, closing factories and forcing workers to stay at home or huge layoffs are not actions policymakers do much. As this crisis is ongoing, the unemployment rate will be higher and the supply chain disrupted further. The main priority of organizations will be the safety to protect their employees and how to maintain business operations in the post-pandemic world. In this context, actions would be taken such as the implementation of a physical mechanism to reduce transmissions like cleaning, staggering shift, and contingency plans for workplace closures. On the other hand, organizations will try to find alternative supply chain actions to respond to the crisis such as transparency on the multi-tier supply chain, optimizing production and distribution capacity, estimating realistic final consumer demand, estimating inventory along the value chain, and finding available logistics capacity [37]. 

As a conclusion, it is expected that pandemic will provide some economic and political opportunities to some countries if they manage to end the virus earlier. According to the discussions presented in this study, we summarize possible economic threats and opportunities for the post-pandemic world in Table. It seems that the Turkish economy will be destroyed in the short term like the many other countries but if Turkey can manage to control the virus soon, this would bring sustainable growth with the accelerating rise in manufacturing exports, tourism revenue and foreign investment in the short term. In addition, the world will not be the same in the postpandemic era due to the changing sensitivities in the social life or production mechanisms or governance of countries, as stated above. Hence, Turkey must develop new strategies to adjust the postpandemic new world order without much delay to gain advantage or not to be left behind.
